# High dielectric thin films based on barium titanate and cellulose nanofibrils†

**DOI:** 10.1039/c9ra10916a

**Published:** 2020-02-04

**Authors:** Jie Tao, Shun-an Cao, Rui Feng, Yulin Deng

**Affiliations:** School of Power and Mechanical Engineering, Wuhan University Wuhan 430072 China shunancao@163.com; School of Material Science and Engineering, Wuhan University of Technology Wuhan 430070 China; School of Chemical & Biomolecular Engineering, Renewable Bioproducts Institute, Georgia Institute of Technology Atlanta GA 30332-0620 USA yulin.deng@rbi.gatech.edu

## Abstract

A series of composite films based on tetragonal barium titanate (BTO) and cellulose nanofibrils (CNF) with high dielectric constant are prepared using a casting method in aqueous solution. No organic solvent is involved during the preparation, which demonstrates the environmental friendliness of the novel material. With less than 30 wt% of filler loading, the excellent distribution of BTO nanoparticles within the CNF matrix is revealed by the FE-SEM images. The dielectric constant of the CNF/BTO (30 wt%) composite film reaches up to 188.03, which is about seven times higher than that of pure CNF (25.24), while the loss tangent only rises slightly from 0.70 to 1.21 (at 1 kHz). The thin films kept their dielectric properties on an acceptable level after repeatedly twisting or rolling 10 times. The improvement of thermal stability is observed with the presence of BTO. The outstanding dielectric properties of the CNF/BTO composite film indicates its great potential to be utilized in energy storage applications.

## Abstract 2

The high dielectric thin films based on cellulose fibrils and tetragonal barium titanate exhibit excellent dielectric properties, flexibility and durability.

## Graphical abstract

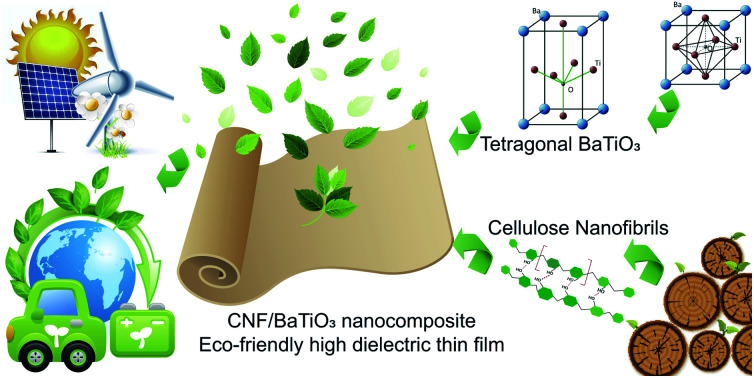

## Introduction

In the past decades, considerable research effort has been made to exploit sustainable energy to replace fossil fuels and reduce pollution.^1^ High dielectric materials (HDMs) are of vital importance in energy storage and conversion devices for solar and wind energy because of their high energy density and power density. HDM based capacitors receive much attention in the applications of portable electronics, hybrid electric vehicles, and so on.^2^ Excellent HDMs can significantly cut down dissipation, improve the efficiency of energy storage, and shrink the size of devices.^3^ Organic/inorganic composites are some of the most extensively studied HDMs as they combine the merits of both components, such as enhanced dielectric properties, outstanding thermal stabilities, bio-compatibility, light weight, low cost and remarkable processing properties.^4–7^

Ferroelectric ceramics such as lead-free barium titanate (BaTiO_3_, BTO) have long been of interest as fillers for high dielectric composites.^8,9^ This is due to their high dielectric constant (as high as 7000),^10^ low dissipation, impressive piezoelectric and pyroelectric properties.^11,12^ The effects of size, shape (*e.g.*, nanoparticles, nanorods, nanowires, nanocubes), crystal phase (*e.g.*, cubic, tetragonal, orthorhombic, rhombohedral), matrix, surface conditions, porosity, filler loading and distributions have been discussed intensively in the literature.^13–18^ By adjusting these factors, a range of BTO based composites have been reported to have splendid dielectric properties.^19–24^ Dang *et al.*^25^ modified BTO particles (diameter = 700 nm) with 1.0 wt% of silane coupling agent (KH550) to improve its compatibility with polyvinylidene fluoride (PVDF), and the relative dielectric constant (*ε*_r_) of the PVDF/BTO (40.0 vol%) composite (thickness = 1 mm) is more than 50, whereas the loss tangent (tan *δ*) is lower than 0.03 (at 1 kHz). In order to improve the thermal stabilities, Dang's research group^26^ then prepared polyimide (PI)/BTO composite by *in situ* polymerization. With 40 vol% of BTO (diameter ∼ 100 nm) loaded, the *ε*_r_ of the films (thickness 10–70 μm) reaches 18, while tan *δ* is below 0.01 (at 1 kHz). More PVDF based polymers are employed as the dielectric matrix, and their *ε*_r_ is in the range from 30 to 50 (at 1 kHz) with tan *δ* lower than 0.05.^27–29^ Kim *et al.*^30^ investigated polycarbonate (PC) as the matrix for chemically functionalized BTO and obtained a series of thin films nanocomposite (thickness = 3.89 μm). With a filler content of 50 vol%, the *ε*_r_ of the sample film is 37 ± 2, and tan *δ* is <0.03 (at 1 kHz). Luo *et al.*^31^ designed a 3D BTO network and synthesized a composite by injecting epoxy solutions into the porous structure. With 30 vol% BTO (size of grain = 0.5–1 μm) loaded, a high *ε*_r_ of 200 is achieved with a tan *δ* of 0.14 (at 1 kHz).

The recent researches based on BTO fillers focus more on improving breakdown strength and reducing tan *δ* to pursue higher energy density and storage efficiency.^31–38^ Generally, most of the applied polymer matrices are non-biodegradable or even toxic. And some of the matrix polymers, such as polycarbonate, reported in the literature are fragile or stiff, which are not suitable for soft and portable electronic applications. Therefore, more and more researchers are exploring safe, green and environmentally friendly alternatives. Cellulose, nanocellulose and their derivatives are reported to be a promising replacement for the traditional polymer dielectric matrices.^39–62^ Chiang *et al.*^63^ mixed BTO (diameter < 2 μm) with cyanoethyl cellulose (CEC) in acetone and prepared a well-dispersed composite with a thickness of 100–350 μm. The *ε*_r_ of the composite increases from 21 for pure CEC to 133 (at 1 kHz) with the addition of 51 vol% BTO. Jia *et al.*^64^ reported a three-phase composite (thickness ∼ 75 μm) based on 30 wt% BTO (diameter ≈ 100 nm), 24 wt% antimony tin oxide (ATO, diameter ≈ 10 nm), and CEC. The *ε*_r_ of the CEC/ATO/BTO nanocomposite film reaches 66.08 with a tan *δ* of ∼0.1 (at 1 kHz). Cellulose is a common and almost inexhaustible resource in nature. Recently, novel nanocellulose garners attention because of its specific properties such as good dielectric properties, low coefficient of thermal expansion, flexible, excellent mechanical properties, high transparency, low density, and strong chemical durability.^65–74^ It worth to note that nanocellulose is made from pure mechanical grinding, thus no toxic solvent is needed for its production. Hassan *et al.*^11^ doped BTO (diameter ≈ 180 nm) into 2,2,6,6-tetramethylpiperidinyl-1-oxyl (TEMPO) oxidized cellulose nanofibrils (CNFs). With 5 wt% BTO added, the *ε*_r_ of the composite films of TEMPO-modified CNF (TCNF) and BTO (thickness of 0.09–1 mm) is 49 000, which is four times higher than that of pure TCNF films (11 034 at 1 kHz). And tan *δ* is increased from ∼0.01 for pure CNF to ∼2. In our previous study, titanium dioxide (TiO_2_) was used as the filler, and the dielectric properties of CNF/TiO_2_ and TCNF/TiO_2_ nanocomposite were compared.^42^ It was found that CNF is a better choice than TCNF as the high dielectric matrix for the sake of reliability and energy saving. With 50 wt% of TiO_2_ loaded, the *ε*_r_ of the CNF/TiO_2_ nanocomposite films is 19.51 with a tan *δ* of 0.81.

Based on the above discussions, here we report a 0–3 composite high dielectric system by directly mixing zero-dimensional BTO with three-dimensional CNF in aqueous solution. A series of flexible free-standing CNT/BTO nanocomposite films was prepared by casting method and hot-press treatment. We choose isotropic BTO particles as filler due to its simplicity synthesis in practical applications. BTO nanoparticles were thermally treated to get a tetragonal phase, which is reported to have a higher *ε*_r_ than a cubic phase.^18^ Theoretically, the positively charged surface of BTO nanoparticles should be able to adhere to the negatively charged surface of CNF.^11^ Since both BTO and CNF are highly dispersible in water, no organic solvent was used during the process. Morphologies, phase compositions, dielectric related properties and thermal stabilities of the CNF/BTO sample films were measured and evaluated, and the results demonstrate that the obtained nanocomposite has great potential to be practically utilized in energy storage applications.

## Experimental

### Materials

CNF slurry with a solid content of ∼3% was supplied by Cellulose Lab Inc. (Canada) and used as received.^75–78^ The CNF has a diameter of 6–80 nm with a length of several micrometers. BaTiO_3_ nanoparticles (diameter < 100 nm, 99.9%) was purchased from Aladdin Co., Ltd. (China), and calcined at 1000 °C for 10 hours. Deionized water was obtained by a Millipore Direct-Q3 water purification system.

### Sample preparation

**Fig. 1:**
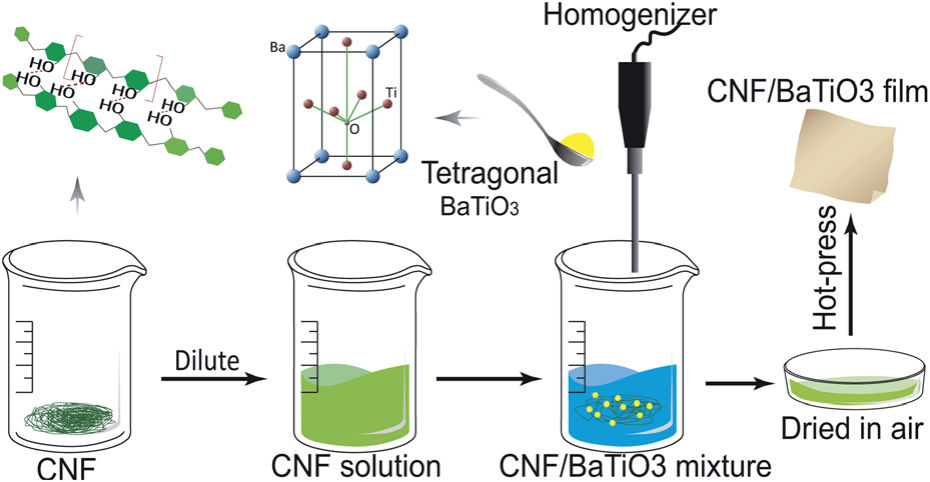
Preparation of CNF/BTO nanocomposite films.

The CNF/BTO nanocomposite films were prepared by simply mixing and solution casting method, as shown in Fig. 1. The CNF/BTO nanocomposite films were prepared by simply mixing and solution casting method, as shown in Fig. 1. CNF slurry was diluted into 0.3 wt% by deionized water, and stirred by a homogenizer (RCD-1A, Changzhou yuexin, China) for 5 min. After the dilution, a certain amount of BTO was added according to the dry weight percentage, and the solution was homogenized for 5 min again. Subsequently, the solution was poured into a Petri dish and dried in a fume hood for several days. Finally, the obtained film was hot-pressed under 80 °C for 3 hours. The thickness of the flexible films was in the range of 30–100 μm.

### Measurement and characterization

The surface and cross-section morphologies of sample films were investigated by field-emission scanning electron microscopy (FE-SEM, TESCAN MIRA3, Czech Republic) with an accelerating voltage of 5 kHz. Prior to the measurement, the samples were coated with a thin layer of gold using a sputter coater (Quorum SC7620, UK) in a vacuum to reduce charge interruptions. Energy-dispersive X-ray spectroscopy (EDS, Oxford X-Max20, UK) was used to analyze the distribution of BTO in the CNF matrix. X-ray diffraction (XRD) patterns of BTO particles and CNF/BTO composite films were determined by an X-ray diffraction spectrometer (XRD, PANalytical XPert Pro, Netherlands). The XRD patterns were recorded with Cu K_α_ radiation (*λ* = 1.542 Å) at 40 kV and 40 mA in the 2 theta (2*θ*) value range from 5° to 80°. The thermal stabilities of the nanocomposites were tested by thermogravimetric analysis and differential scanning calorimetry (TGA-DSC, Netzsch STA449F3, Germany) at a heating rate of 10 °C min^−1^ from 35 °C to 790 °C under a nitrogen atmosphere. The dielectric constant, loss tangent and AC electrical conductivity of sample films were studied using an LCR meter (Keysight E4980A, USA). The results were recorded in the frequency range from 40 Hz to 1 MHz with an oscillation signal of 1 V at ambient temperature. Prior to the measurement, gold electrodes were sputtered on both sides of the specimens, and the test was repeated four times for each composition. The relative dielectric constants (*ε*_r_) of the sample films were calculated by eqn (1):1
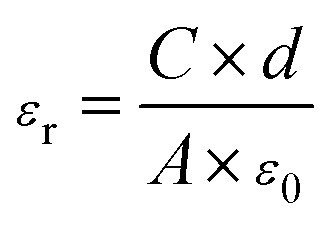
where, *C* is the capacitance; *ε*_0_ is the absolute dielectric constant of vacuum, *ε*_0_ = 8.854 × 10^−12^ F m^−1^; *A* is the electrode area, *A* = 4.52 × 10^−6^ m^2^; *d* is the thickness of the sample film. The polarization–electric field (*P*–*E*) curves of the sample films were measured with a ferroelectric tester (Radiant Precision Multiferroic II, USA) at 100 Hz.

## Results and discussion

### Morphology

**Fig. 2:**
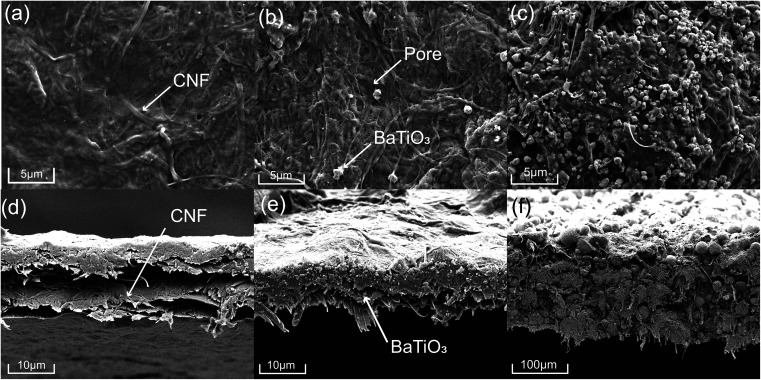
Surface and cross-section FE-SEM micrographs of pure CNF (a and d), CNF/BTO (30 wt%) (b and e) and CNF/BTO (90 wt%) (c and f) composite films.

Well dispersion of BTO filler is conducive to uniform packing of CNF/BTO nanocomposite, leading to better dielectric properties. The surface and cross-section morphologies of the pure CNF and CNF/BTO composite films were investigated by FE-SEM images (shown in Fig. 2). Pure CNF film had a smooth surface and laminated structure (Fig. 2a and d). The morphological changes induced by BTO fillers on the CNF are very clear. In the lower range of filler content, BTO particles exhibited good dispersion in the CNF matrix, in turn, the composite films kept as dense as pure CNF film. With the addition of 30 wt% BTO, small voids and a rough surface started to be seen due to the aggregation of BTO (Fig. 2b and e). BTO particles with a diameter of 0.5–1.0 μm are resulted from high-temperature calcination, and there are strong forces between particles.^79^ Generally, BTO particles are still uniformly dispersed throughout the CNF matrix, which is in good accordance with EDS results (Fig. S1–S6†). In the specimens with higher BTO content, the laminated structure disappeared, and big spheres with a diameter of >10 μm are observed (Fig. 2c and f). The films exhibit porous and loose structure due to the weakening of CNF chain–chain interactions by the inclusion of BTO particles.^79^ This result shows that uniform CNF/BTO nanocomposite films can be obtained by simple mechanical mixing and casting methods when filler content below 30 wt%.

### Phase composition

**Fig. 3:**
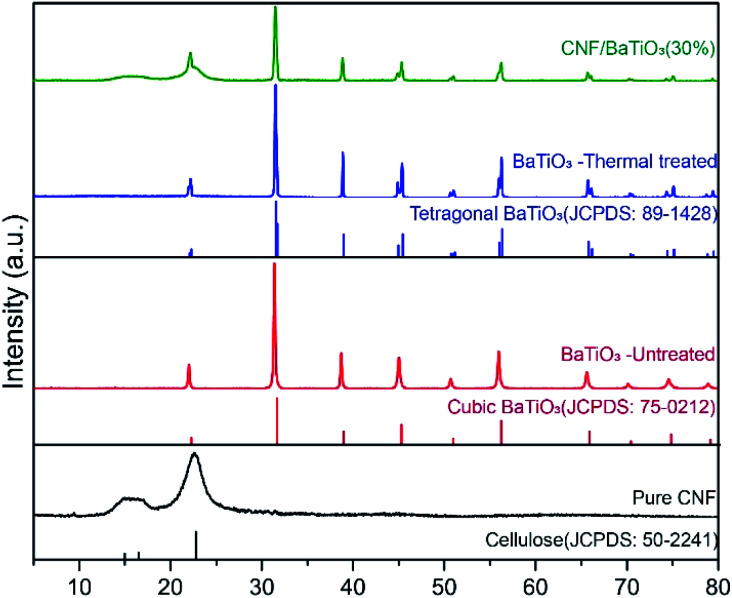
XRD patterns of pure CNF, untreated BTO, thermally treated BTO, and CNF/BTO (30 wt%) nanocomposite films.

The dielectric properties of the composite are influenced by the crystal properties of the constituents. Tetragonal BTO has a higher dielectric constant than other phases, *e.g.*, cubic phase. Taking this advantage, tetragonal BTO has much higher commercial value than other phases. The phase compositions of pure CNF, untreated BTO, thermally treated BTO, and CNF/BTO (30 wt%) composites were recorded by XRD patterns, as is presented in Fig. 3. The abroad peak in the region of 15° to 17° and at 22.6° are corresponding to the partly crystalline structure of CNF.^11,62,80^ The peak intensity of CNF in the nanocomposite films decreased with the addition of BTO due to the destruction of the ordered structure.^64^ The XRD pattern of thermally treated BTO was in line with tetragonal BTO (JCPDS No. 89-1428). Especially, the characteristic splitting peak of the tetragonal phase was seen distinctively at 45°.^79,81,82^ Owing to the higher crystallinity of BTO, its peaks were much sharper than that of CNF. In the XRD pattern of CNF/BTO composite, the peak intensity of both CNF and BTO decreased compared with pure CNF and BTO. It is ascribed to the addition of nanofillers, which destroys the ordered structure of the CNF and reduces its crystallinity.^83^ It is worth mentioning that the peak positions of both CNF and BTO remained unchanged in the CNF/BTO composite, which indicates that the mechanical mixing process does not affect the crystalline structure of these two constitutes.^61^

### Dielectric properties

**Fig. 4:**
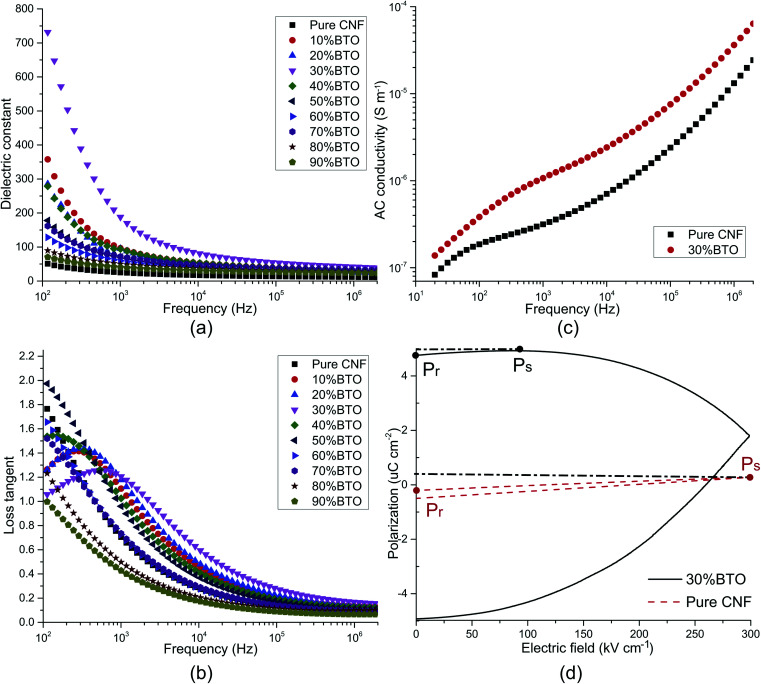
Dielectric properties of CNF/BTO nanocomposite films. The influence of frequency and filler content on the (a) dielectric constant, (b) dielectric loss, and (c) AC conductivity (d) *P*–*E* hysteresis curves of CNF/BTO nanocomposites.

The dielectric properties usually refer to the relative dielectric constant (*ε*_r_) and loss tangent (dissipation factor, tan *δ*). Towards the application of energy storage, conductivity and *P*–*E* hysteresis loop were also examined. All of these properties are considerably affected by the frequency. The frequency dependence of *ε*_r_ and tan *δ* was studied, as shown in Fig. 4a and b. In the low-frequency range of 40–10 kHz, both *ε*_r_ and tan *δ* decreased sharply with the increase of frequency, and a tan *δ* peak was presented in the plot of samples with 0–40 wt% BTO. These are typical characteristics of electrode effect and Maxwell–Wagner–Sillars interfacial polarization.^50,60,84–86^ With the frequency going up, the high periodic reversal of the electric field makes it harder for dipoles and charge carriers, which accumulated at the interface, to catch up with the change of frequency. Hence, the interfacial polarization is decreased and results in lower *ε*_r_ and tan *δ*.^87^

In the meantime, *ε*_r_ increases with filler content before 30 wt% and decreases with the further addition of BTO. It can be explained by Tanaka's model, which assumes that the interface of a filler particle consists of three layers: a bonded layer, a bound layer, and a loose layer. Additionally, there is an electric double layer overlapping the above three layers.^88^ Reducing the particle size of BTO to the nanoscale, as well as increasing BTO loading, offers larger surface areas for interfacial polarization, resulting in an extraordinary increase of both *ε*_r_ and tan *δ*.^72^ However, at the same time, the bonded layer and bound layer of the nanoparticles impair the motion of dipoles and leads to a decrease of *ε*_r_ and tan *δ*. Besides, the response of dipoles and ionic carriers in the loose layer is more complicated. Coincidentally, BTO aggregations started to be seen with a filler content of 30 wt% in FE-SEM images. It can be ascribed to the far-field effect of the double electric layer, which makes the loose layers of neighbored nanoparticles collaborate with each other. On the one hand, the resulted imperfection of heterogeneous structures helps to improve *ε*_r_ and tan *δ*. On the other hand, the porous structure brought by severe aggregation can dramatically cut down *ε*_r_ and tan *δ*.

In the higher frequency range of above 100 kHz, *ε*_r_ and tan *δ* were almost insusceptible to the frequency and remains constant. It illustrates that electronic polarization, atomic polarization, and orientation polarization starts to play a predominant role. In this frequency range, filler content also has no obvious influence on *ε*_r_ and tan *δ*. The *ε*_r_ of CNF/BTO composite films reaches its maximum value of 188.03 with 30 wt% of BTO, which is more than seven times higher than that of pure CNF, whose *ε*_r_ is 25.24 (at 1 kHz). Meanwhile, the tan *δ* is only slightly grown from 0.70 to 1.21 (at 1 kHz). The dielectric constant of CNF/BTO composite is competitive with other BTO based composites, whose *ε*_r_ is in the range from 30 to 50,^25−30^ and cellulose-based composites, whose *ε*_r_ is lower than 135.^63,64^ However, similar to most of other dielectric composites, higher *ε*_r_ is accompanied with higher tan *δ*. Therefore, more efforts to decrease the loss tangent is still needed. In general, the outstanding dielectric properties of CNF/BTO nanocomposite film render it a promising candidate for capacitor applications.

To further explore the electric properties of CNF/BTO (30 wt%) nanocomposites, the frequency dependence of AC conductivity (*σ*_AC_) was tested and compared with pure CNF (as shown in Fig. 4c). The *σ*_AC_ of both samples ascended with the increase of frequency. In the lower frequency range of 40 Hz to 100 kHz, the trend was gradual, while it turned rapidly with the frequency further increased. This phenomenon complies well with Dyre's random free energy barrier model.^89^ The frequency-dependent property of *σ*_AC_ is related to the hopping of the charge carriers in the localized state and upper states in the conduction band. And this process can be accelerated by higher frequency.^11,61^ Composite films with 30 wt% BTO exhibited higher *σ*_AC_ than that of pure CNF. It is attributed to its higher crystallinity, as evidenced by XRD patterns. Besides, the integration of BTO in CNF piles up more interfacial areas and charges, resulting in higher *σ*_AC_. At 1 kHz, the *σ*_AC_ increased from 3.15 × 10^−7^ S cm^−1^ for CNF to 1.07 × 10^−6^ S cm^−1^ for CNF/BTO (30 wt%).

The polarization–electric field (*P*–*E*) hysteresis curve is an efficient way to evaluate the energy storage properties. The *P*–*E* hysteresis curves of pure CNF and the CNF/BTO (30 wt%) were measured, as shown in Fig. 4d. Both remnant polarization (*P*_r_) and saturated polarization (*P*_s_) of CNF/BTO composite films were higher than that of pure CNF. It demonstrates that the addition of BTO makes it easier to be polarized, which is also proved by the results that CNF/BTO composite has a higher *ε*_r_ than that of pure CNF. However, the *P*_r_ of the composite film was very close to *P*_s_, suggesting a low efficiency of energy storage.^11^ The polarization of CNF/BTO film saturated before the electric field reached its maximum value. It may be caused by the leakage current which is the result of a heterogeneous structure. In order to be utilized in the area of energy storage application, more efforts should be made on improving the dispersion of BTO fillers and reducing the remnant polarization.

### Influence of twisting treatments

**Fig. 5:**
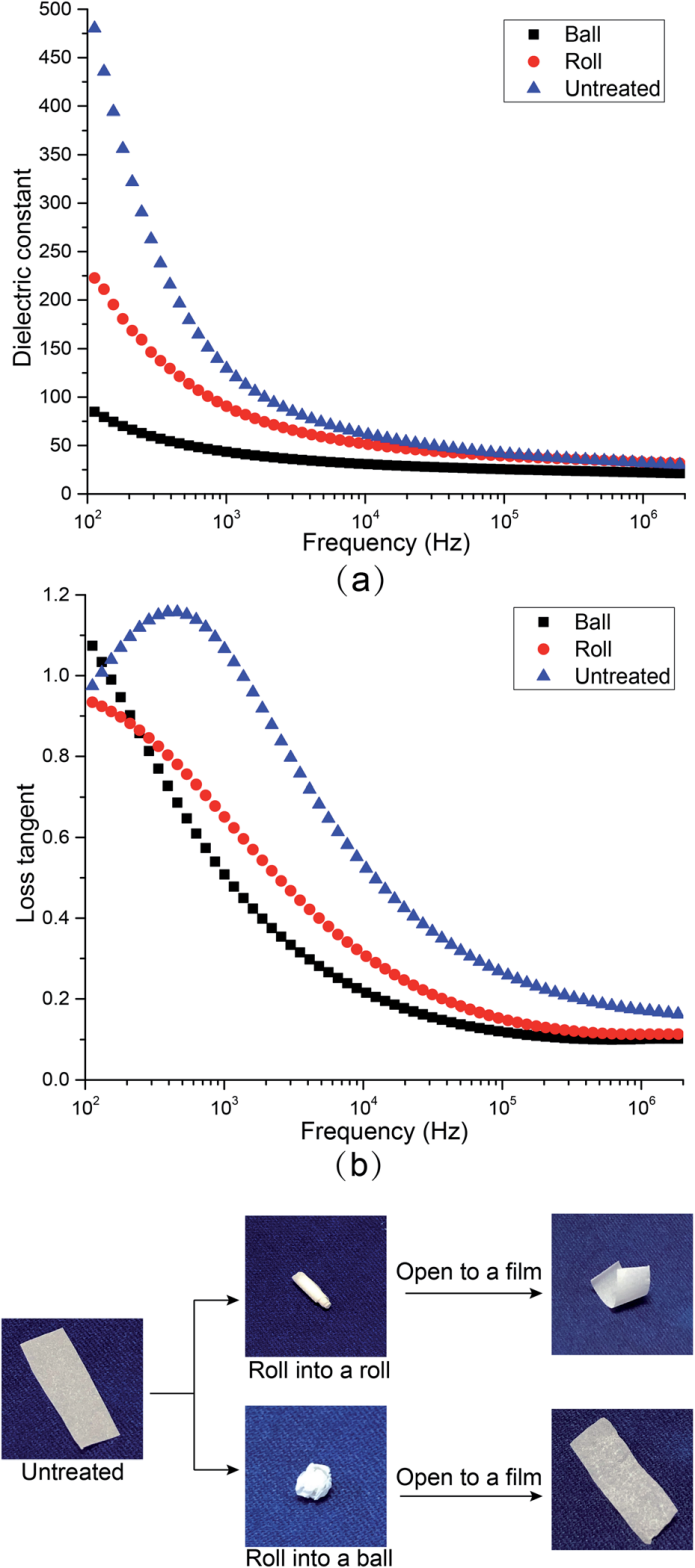
The influences of rolling treatments on (a) dielectric constant and (b) loss tangent of CNF/BTO nanocomposite films.

In order to study the potential of this high dielectric film as a soft electronic material, the dielectric properties of CNF/BTO (30 wt%) nanocomposite after being repeatedly twisted into a roll (CNF/BTO-R) and ball (CNF/BTO-B) for 10 times, and their dielectric properties were measured again, as shown in Fig. 5. Though both the *ε*_r_ and tan *δ* decreased after the rolling treatment. The *ε*_r_ for untreated CNF/BTO, CNF/BTO-R, and CNF/BTO-B are 129.56, 90.58, and 43.44, respectively, while the tan *δ* are 1.07, 0.65, and 0.51, respectively. The CNF/BTO-B films exhibited lower *ε*_r_ and tan *δ* than that of CNF/BTO-R, which might be caused by more air being entrapped in the film during the twisting or rolling process. This was proved by the observation of the rougher surface and higher thickness (by ∼5%) of CNF/BTO-B. It is worth to note that the rolled CNF/BTO-B films still showed higher dielectric properties compared with other reported composites.^42^ And at high frequency, the difference of *ε*_r_ and tan *δ* between these samples are much smaller, which suggests the possibility of the flexible CNF/BTO film to be utilized in the high-frequency applications such as antenna.

### Thermal stability

**Fig. 6:**
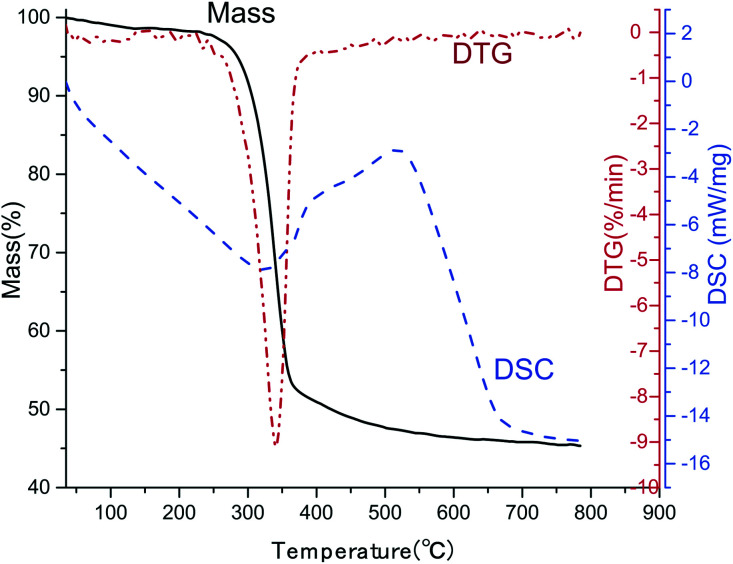
TGA-DSC curves of CNF/BTO (30 wt%) nanocomposite film.

In practical application, thermal stability is one of the most important concerns. The TGA-DSC curve of CNF/BTO (30 wt%) composite film was measured, as depicted in Fig. 6. Two steps were presented in the degradation process. The first degradation step is the slight weight loss before 100 °C, resulting from the evaporation of moisture.^42^ The temperature at 5.0% weight loss (*T*_5%_) is considered as the start signal of degradation. CNF/BTO (30 wt%) nanocomposite has a *T*_5%_ of 284 °C, which is slightly higher than that of pure CNF (265 °C, Fig. SI 7†). It means that the doping of BTO makes an improvement in thermal stability. The second degradation step located in the temperature range from 250 °C to 400 °C, where there is a drastic drop of weight, combined with a DTG peak at 340 °C and a DSC endothermal peak at 325 °C. This dramatic drop is due to the decomposition of CNF.

## Conclusions

A series of high dielectric nanocomposite films based on zero-dimensional tetragonal BTO and three-dimensional CNF were prepared by casting method in aqueous solution. No organic solvents were involved during the preparation, which illustrates the environmental friendliness of the composites. BTO had good distribution within the CNF matrix in the lower range of filler content. The high amount of BTO particles resulted in a porous structure, which led to the drop of dielectric constant. The *ε*_r_ of CNF/BTO composite films reached its maximum value of 188.03 with 30 wt% of BTO, which was more than seven times higher than that of pure CNF, whose *ε*_r_ was 25.24 (at 1 kHz). Meanwhile, the tan *δ* was only slightly grown from 0.70 to 1.21 (at 1 kHz). The thin film keeps its dielectric properties on an acceptable level even after the twisting treatments. The presence of BTO helps to improve the thermal stability of the composite films. More efforts still need to be made to cut down the remanent polarization and improve the compatibility between the BTO filler and CNF matrix. However, the excellent properties of CNF/BTO composite film demonstrate its great potential to be utilized as a green candidate for HDMs in the field of energy storage.

## Conflicts of interest

There are no conflicts to declare.

## Supplementary Material

RA-010-C9RA10916A-s001
