# Supplementary material for: High dielectric thin films based on barium titanate and cellulose nanofibrils
Source: RSC Adv. 2020 Feb 4;10(10):5758–65. doi: 10.1039/c9ra10916a (PMC9049558; doi:10.1039/c9ra10916a)

Electronic Supplementary Material (ESI) for RSC Advances.
This journal is © The Royal Society of Chemistry 2020

Supporting Information

High Dielectric Thin Films Based on Barium Titanate and Cellulose Nanofibrils

Jie Tao, Shun-an Cao, Rui Feng, and Yulin Deng**

Fig. S1 Energy-dispersive X-ray spectroscopy of CNF/BTO (30 wt.%)- EDS layered image

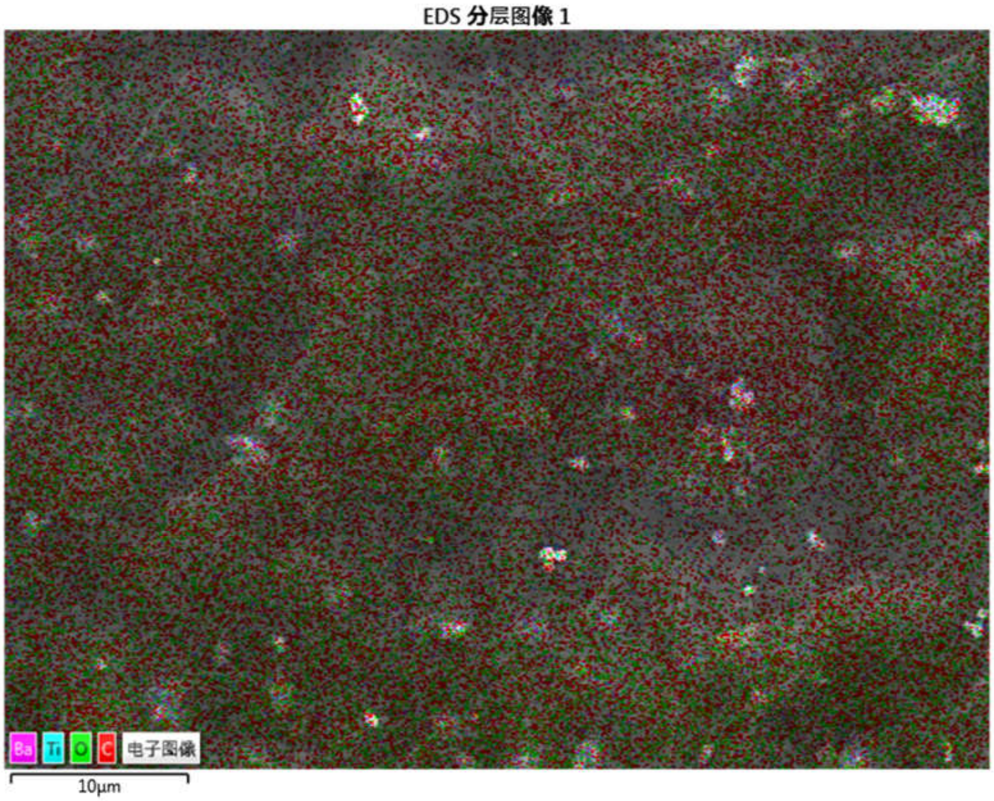

Fig. S2 EDS electronic image of CNF/BTO (30 wt.%)

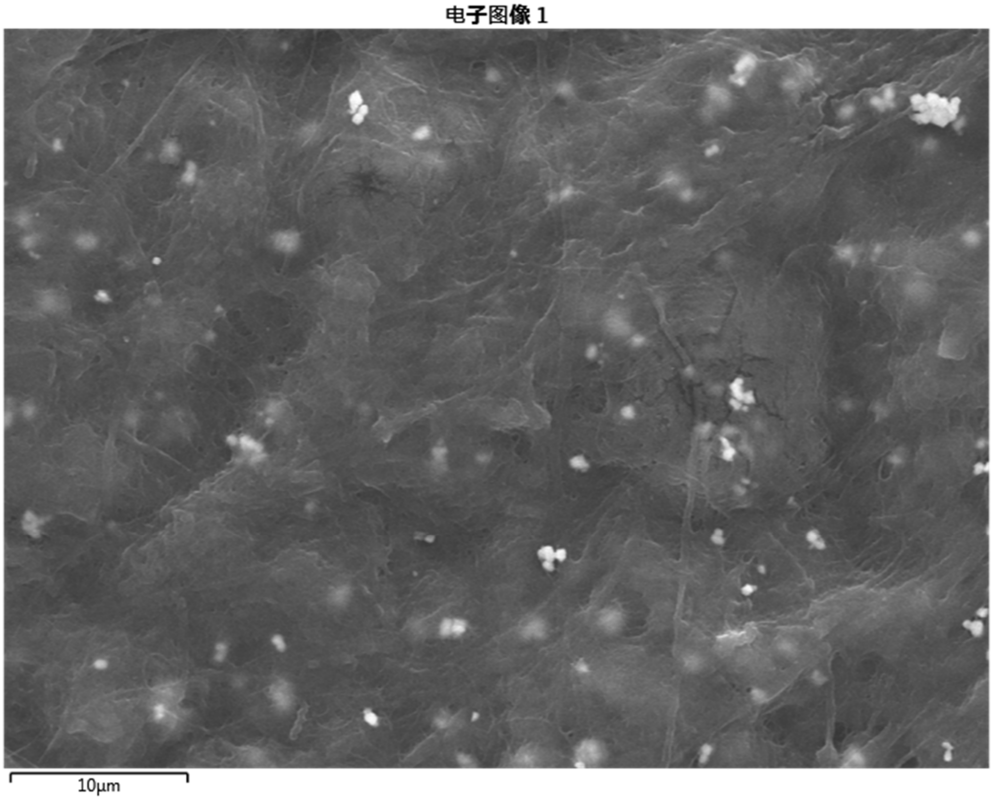

Fig. S3 EDS C Kα1_2 image of CNF/BTO (30 wt.%)

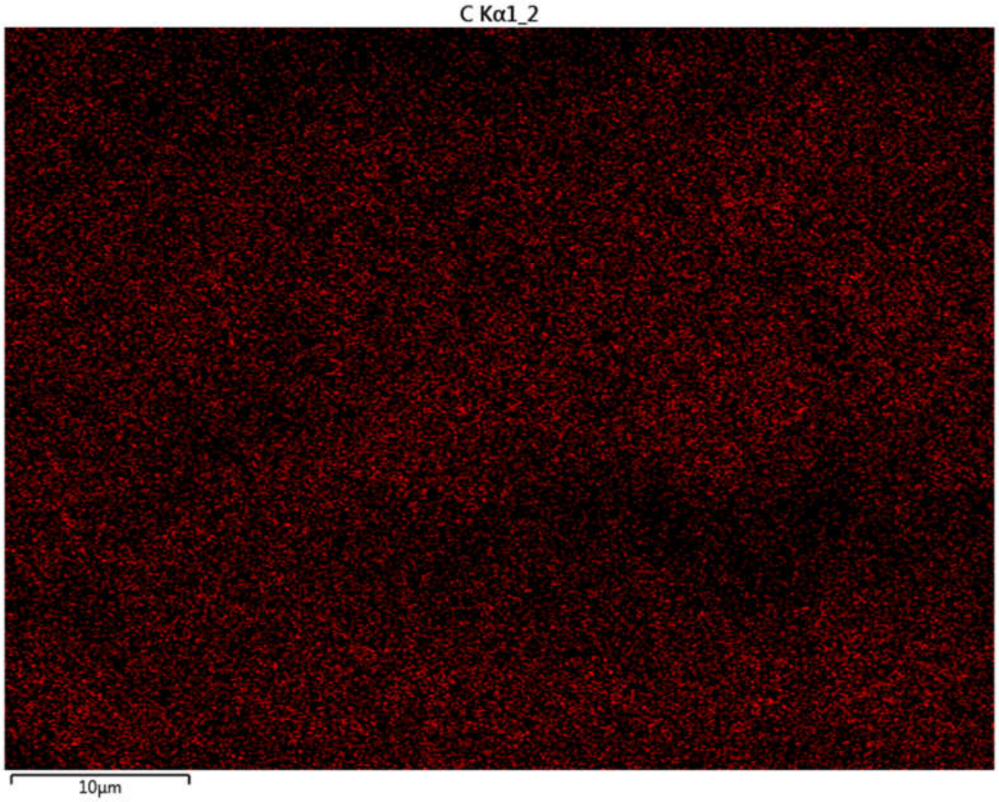

Fig. S4 EDS O Kα1 image of CNF/BTO (30 wt.%)

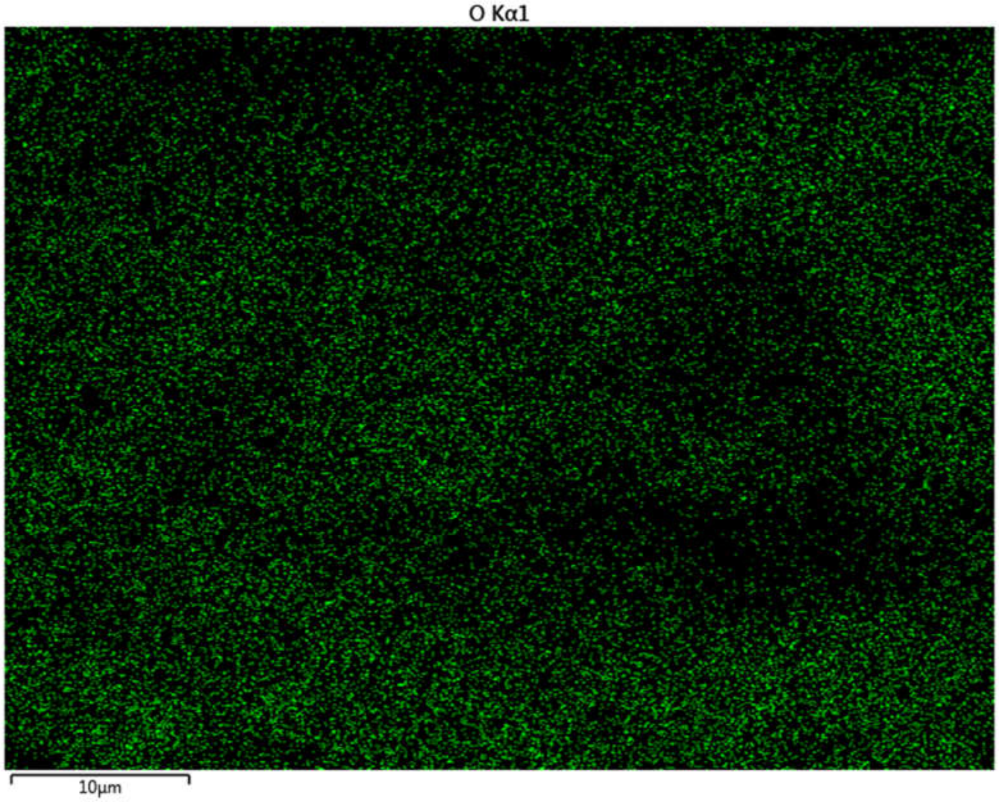

Fig. S5 EDS Ti Kα1 image of CNF/BTO (30 wt.%)

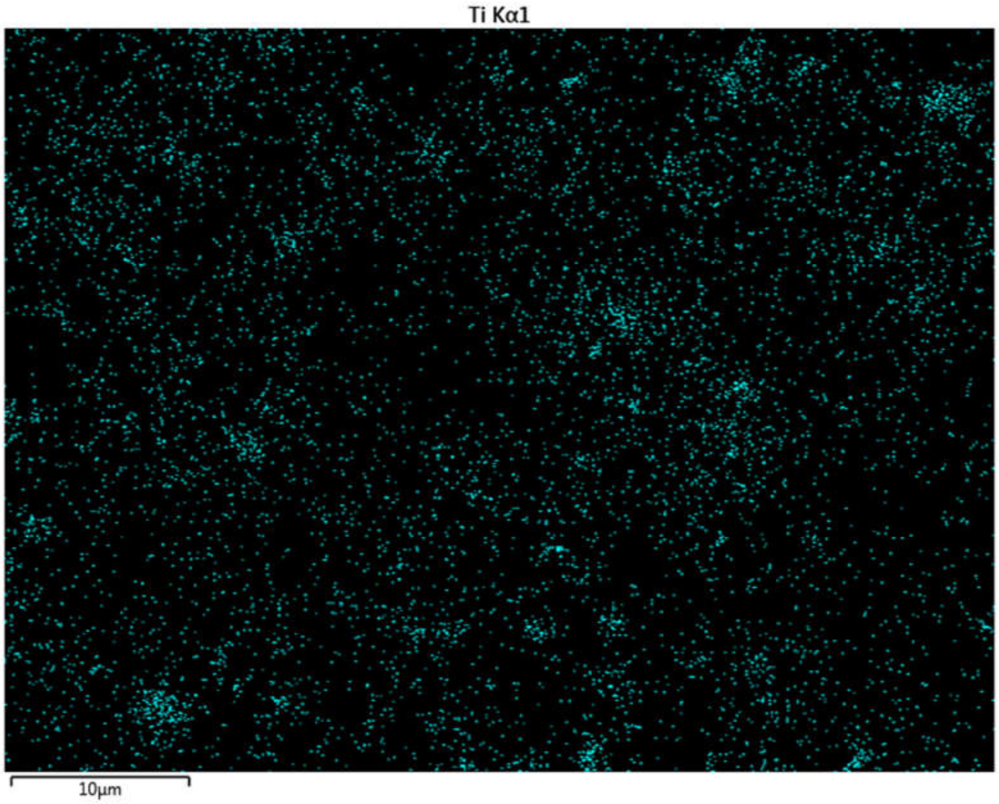

Fig. S6 EDS Ba Lα1 image of CNF/BTO (30 wt.%)

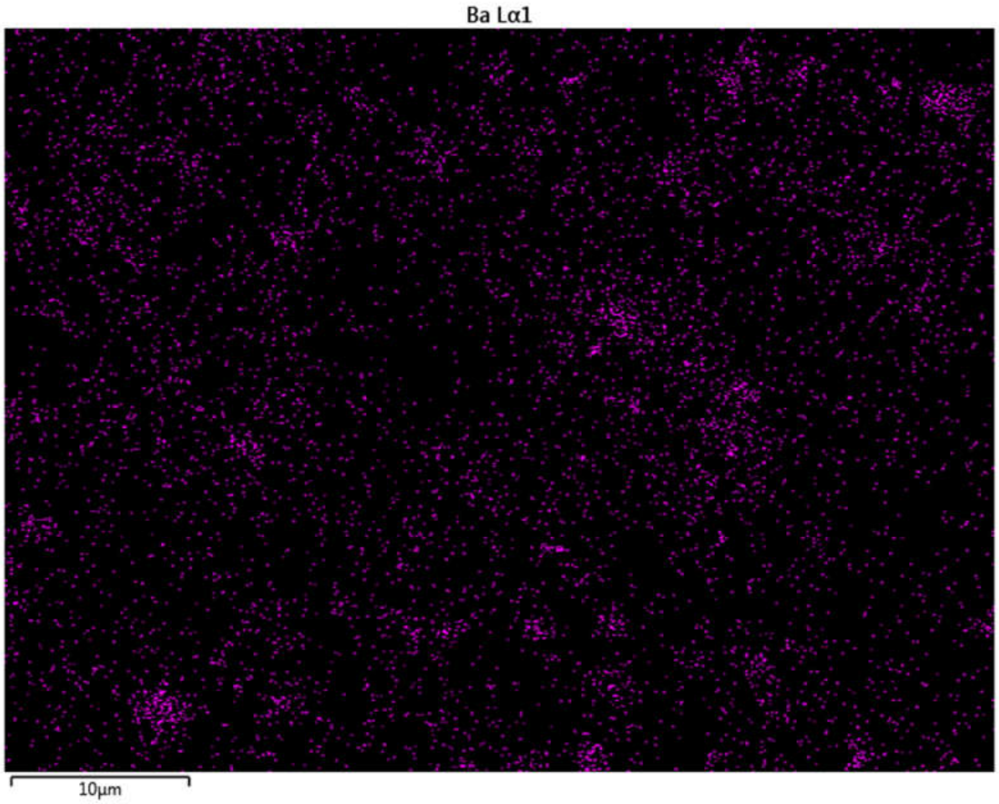

Fig. S7 TGA-DSC curves of pure CNF

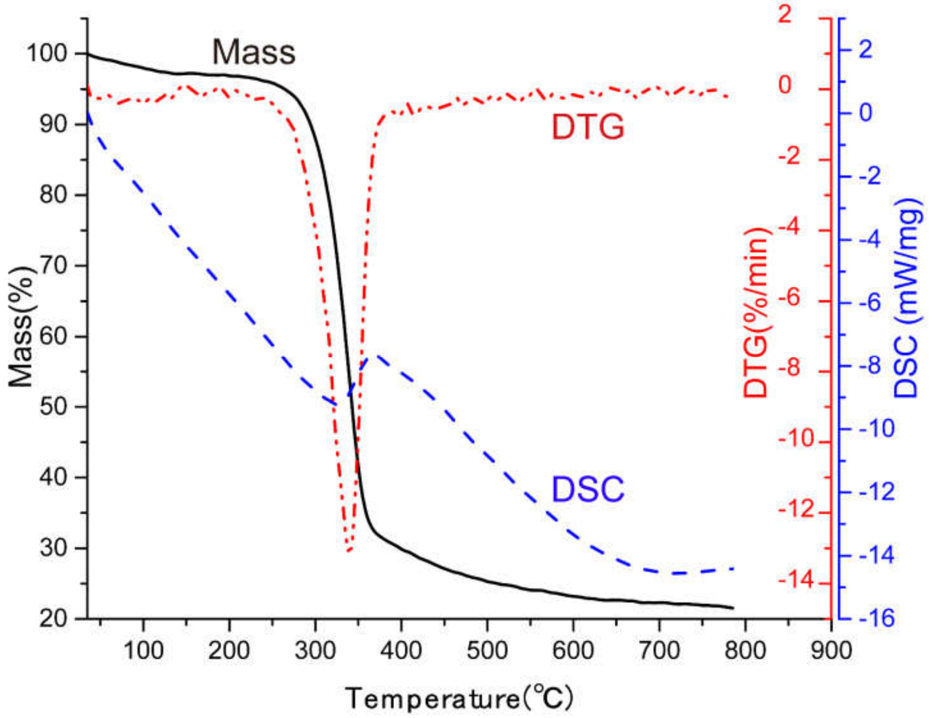

Supplement: RA-010-C9RA10916A-s001 [file RA-010-C9RA10916A-s001.pdf]
